# The PLAC1-homology region of the ZP domain is sufficient for protein polymerisation

**DOI:** 10.1186/1471-2091-7-11

**Published:** 2006-04-06

**Authors:** Luca Jovine, William G Janssen, Eveline S Litscher, Paul M Wassarman

**Affiliations:** 1 Brookdale Department of Molecular, Cell and Developmental Biology, Mount Sinai School of Medicine, One Gustave L. Levy Place, New York, NY 10029-6574, USA; 2 Department of Neuroscience, Mount Sinai School of Medicine, One Gustave L. Levy Place, New York, NY 10029-6574, USA; 3 Department of Biosciences and Nutrition, Center for Structural Biochemistry, Karolinska Institutet, Hälsovägen 7, Huddinge S-141 57, Sweden

## Abstract

**Background:**

Hundreds of extracellular proteins polymerise into filaments and matrices by using zona pellucida (ZP) domains. ZP domain proteins perform highly diverse functions, ranging from structural to receptorial, and mutations in their genes are responsible for a number of severe human diseases. Recently, PLAC1, Oosp1-3, Papillote and CG16798 proteins were identified that share sequence homology with the N-terminal half of the ZP domain (ZP-N), but not with its C-terminal half (ZP-C). The functional significance of this partial conservation is unknown.

**Results:**

By exploiting a highly engineered bacterial strain, we expressed in soluble form the PLAC1-homology region of mammalian sperm receptor ZP3 as a fusion to maltose binding protein. Mass spectrometry showed that the 4 conserved Cys residues within the ZP-N moiety of the fusion protein adopt the same disulfide bond connectivity as in full-length native ZP3, indicating that it is correctly folded, and electron microscopy and biochemical analyses revealed that it assembles into filaments.

**Conclusion:**

These findings provide a function for PLAC1-like proteins and, by showing that ZP-N is a biologically active folding unit, prompt a re-evaluation of the architecture of the ZP domain and its polymers. Furthermore, they suggest that ZP-C might play a regulatory role in the assembly of ZP domain protein complexes.

## Background

The ZP domain is a sequence of ~260 amino acids that drives polymerisation of a large number of essential secreted proteins from multicellular eukaryotes [1-3]. It has been suggested that the domain, which includes 8 highly conserved Cys residues, consists of two subdomains [4-6]. The N-terminal subdomain (ZP-N) is thought to contain conserved Cys 1 to 4, disulfide-bonded with invariant 1–4, 2–3 connectivity. On the other hand, conserved Cys 5 to 8, located within the C-terminal subdomain (ZP-C), apparently adopt two alternative connectivities in different ZP domain proteins [3,6-10]. In type I ZP domain proteins with 8 Cys within the ZP domain, such as ZP3, the ZP-C connectivity is 5–7, 6–8; in type II ZP domain proteins with 10 Cys within the ZP domain, like the other egg coat subunits ZP1 and ZP2, it is 5–6, 7-*a*, *b*-8 (*a *and *b *being the two additional Cys, compared to type I proteins). Interestingly, type I (ZP3-like) ZP domain proteins appear to polymerise into filaments only in the presence of type II (ZP1/ZP2-like) ZP domain proteins, whereas the latter can also form homopolymers.

Recently, placenta protein PLAC1 was described that bears significant homology to the N-terminal subdomain of sperm receptor ZP3 [11,12]. Based on this similarity, as well as on the observation that deletion of the X chromosome region harboring the *PLAC1 *gene causes fetal growth restriction and abnormal placenta development [13,14], it was proposed that PLAC1 might be required for interaction between the trophoblast and other placental or maternal tissues [11,15]. Five additional proteins, mammalian Oosp1-3 and *Drosophila *Papillote and CG16798, were subsequently identified that also share homology with ZP-N, but not ZP-C [16-19]. In view of the higher structural conservation of ZP-N, these reports raise questions about the relative contribution of the two subdomains to ZP domain function. Are PLAC1-like proteins also able to polymerise, or do ZP-N sequences carry out a different role than complete ZP domains?

## Results

### Identification of additional protein sequences containing only ZP-N

**Figure 1:**
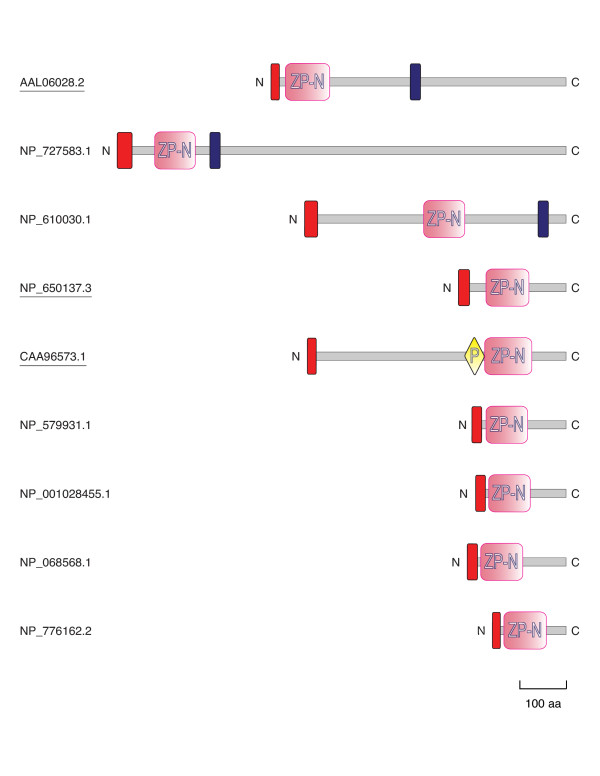
**Architecture of ZP-N-containing proteins**. The primary sequence of each protein is shown as a grey bar, drawn to scale and with the amino and carboxy termini marked. Signal peptides (as identified by SignalP) and transmembrane domains (as predicted by SMART) are represented by red and blue rectangles, respectively; ZP-N sequences are shown as pink rectangles and a trefoil (P) domain is depicted as a yellow rhombus. Proteins are in the same order as in Table 1 and identified by their accession number.

To investigate whether other proteins exist that contain only the N-terminal half of the ZP domain, we generated a profile hidden Markov model (HMM) of ZP-N to scan genomic and non-redundant sequence databases. This analysis identified three additional putative ZP-N-containing proteins, whose genes appear to be expressed (Table 1 and Fig. 1, underlined sequences). On the other hand, no proteins containing only ZP-C were found in a parallel search with a corresponding HMM profile. These observations suggest that, unlike ZP-N, ZP-C can be found exclusively within the context of a complete ZP domain.

**Table 1:** ZP-N proteins

**Species**	**Protein name (accession number)**	**Amino acid number**	HMM search*	**Signal peptide**^†^	**Expression evidence**	**Reference(s)**	**Putative homolog(s)**
*C. elegans*	F55A4.10 (AAL06028.2)	633	3.7e-13; 62.3; 31–126 (4)	0.999 (1–18); 11.327 (1–18)	ESTs (AU201804, CB402430), microarray (WormBase WBGene00018861)	[56]	-
*D. melanogaster*	Papillote/CG2467 (NP_727583.1)	963	4e-15; 69.0; 80–167 (4)	0.999 (1–32); 11.890 (1–32)	mRNA (AY862156), immunohisto-chemistry and Western blot [16], *in situ *hybridization (BDGP CG2467; [17])	[16, 17]	EAL32136.1 (*D. pseudoobscura*)
*D. melanogaster*	CG16798 (NP_610030.1)	561	9.9e-12; 57.7; 255–343 (4)	1.000 (1–28); 13.662 (1–28)	mRNA (AY122225), *in situ *hybridization (BDGP CG16798; [17])	[17]	SNAP00000007590 (*A. gambiae*)
*D. melanogaster*	CG10005 (NP_650137.3)	231	4.3e-17; 75.5; 59–151 (4)	0.999 (1–24); 6.625 (1–19)	mRNA (AY113516), microarray (BDGP CG10005)	-	EAL28621.1 (*D. pseudoobscura*), SNAP00000007531 (*A. gambiae*)
*C. carpio*	ZP1^§^(CAA96573.1)	555	8.4e-29; 114.4; 381–482 (4)	0.999 (1–19); 6.833 (1–19)	MRNA (Z72492), Northern blot, *in situ *hydridization, immuno-histochemistry, Western blot [41]	[41]	-
*M. musculus*	Oosp1 (NP_579931.1)	202	3.2e-07; 42.7; 30–119 (4)	1.000 (1–21); 8.733 (1–21)	mRNA (AF420487), Northern blot, *in situ *hydridization [18]	[18, 19]	ENSRNOP00000028498 (*R. norvegicus*)
*M. musculus*	LOC225923/Oosp3 (NP_001028455.1)	194	1.2; 19.9; 28–117 (4)	1.000 (1–21); 8.275 (1–21)	mRNA (NM_001033283), RT-PCR, *in situ *hybridization [19]	[19]	-
*H. sapiens*	PLAC1 (NP_068568.1)	212	8.7e-11; 54.6; 29–119 (4)	0.999 (1–22); 7.539 (1–23)	MRNA (BC022335), Northern blot, *in situ *hydridizationm [11, 12, 15]	[11, 12, 15]	ENSPTRP00000038397 (*P. troglodytes*), ENSBTAP00000008260 (*B. taurus*), ENSCAFP00000027834 (*C. familiaris*), NP_001020065 (*R. norvegicus*), NP_062411.1 (*M. musculus*)
*H. sapiens*	LOC219990/Oosp2 (NP_776162.2)	158	0.0067; 28.4; 25–116 (4)	0.997 (1–17); 10.702 (1–17)	MRNA (NM_173801)	[19, 57]	ENSPTRP00000006381 (*P. troglodytes*) NP_001032723.1 (*M. musculus*)

* E-value; bit score; matched aa (number of Cys). Calibrated expectation values are relative to the NCBI non-redundant protein database (2308679 sequences at the time of the search).

^†^SignalP probability (aa); SigCleave score (aa).

^§^This protein is referred to as ZP2 in ref. 41. However, because it contains a trefoil domain immediately before the ZP domain, it should be regarded as a member of the ZP1 family.

### Expression, purification and characterisation of recombinant ZP-N

**Figure 2:**
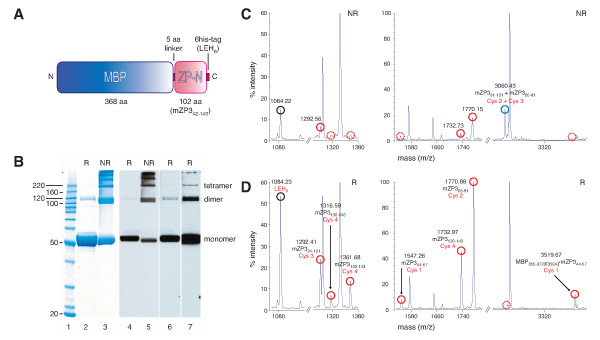
**Characterisation of MBP-ZP-N-6his**. **(A) **Schematic representation of MBP-ZP-N-6his fusion protein. **(B) **Multimerisation of MBP-ZP-N-6his. Purified protein, separated by SDS-PAGE under both reducing (R, lanes 2, 4, 6 and 7) and non-reducing (NR, lanes 3 and 5) conditions, was visualised by Coomassie staining (lanes 2, 3) and by immunoblot analysis with monoclonal anti-6his (lanes 4–7). Lane 1, M_r _markers; lanes 6 and 7, progressively long exposures of lane 4. The position of bands corresponding to monomeric, dimeric and tetrameric MBP-ZP-N-6his is indicated. **(C, D) **Disulfide linkages of monomeric MBP-ZP-N-6his. The fusion protein contains 4 Cys residues, all within the ZP-N sequence. Native 1–4, 2–3 disulfides were assigned on the basis of MALDI-TOF-MS measurements of trypsin-digested MBP-ZP-N-6his, performed under non-reducing (NR, C) and reducing (R, D) conditions (Methods). MBP and ZP3 amino acid numbers refer to database entries 1HSJ_A and P10761, respectively. Peaks represent average mass/charge ratio (m/z). Disulfide-bonded and free Cys-residue containing peptides are marked by blue and red circles, respectively; LEH_6 _C-terminal tag peptide is marked by a black circle; peaks with intensity below 5% are indicated by dashed circles.

To establish whether ZP-N is able to fold independently and investigate its biological role, we over-produced in recombinant form the PLAC1-homology region of the ZP domain of mouse ZP3. The 102-amino acid ZP-N fragment was expressed as an affinity sandwich [20], with *E. coli *maltose binding protein (MBP) fused to its N-terminus via a short linker and a polyhistidine tag (6his) fused to its C-terminus (Fig. 2A). MBP was chosen as a fusion partner since it is strictly monomeric in the presence of maltose [21,22] and has either no or minimal interaction with the proteins to which it is fused, so that the stoichiometry of MBP fusion proteins is entirely determined by the properties of the non-MBP moieties [22,23].

Using a bacterial strain that facilitates formation of disulfides by carrying *trxB *and *gor *mutations [24] and co-expressing modified versions of disulfide isomerase [24] and thioredoxin [25], significant amounts of MBP-ZP-N-6his were obtained that could be purified to homogeneity with a two-step affinity method (Fig. 2B, lane 2).

Although the fusion protein was soluble, as judged by ultracentrifugation at 100,000 g, it eluted in the void volume of 300 kDa molecular weight (M_r_) cut-off size-exclusion columns, suggesting the presence of multimers. Analysis in the presence of ethylenedinitrilotetraacetic acid (EDTA) yielded identical elution profiles, excluding the possibility that trace amounts of Ni^2+ ^ions could have leaked from the immobilised metal ion affinity chromatography (IMAC) column used during purification and caused non-specific protein aggregation by cross-linking multiple histidine tags.

Western blot analysis of purified MBP-ZP-N-6his revealed a band corresponding to monomeric protein and, in addition, a ladder of bands corresponding to dimers, tetramers etc. (i.e. 2n × M_r_, with n = 1, 2, ...) (Fig. 2B). Although these multimers were much less abundant under reducing conditions, several lines of evidence suggest that this was due to more extensive denaturation of the ZP domain moiety of MBP-ZP-N-6his, rather than to the presence of spurious intermolecular disulfides. First, unlike the situation reported for other proteins [26], no bands were observed for trimeric, pentameric, etc. (i.e. (2n+1) × M_r_) forms of MBP-ZP-N-6his (Fig. 2B). Second, as seen in the case of bands corresponding to the monomeric protein, dimeric and tetrameric MBP-ZP-N-6his also migrated differently under reducing and non-reducing conditions (Fig. 2B, compare lanes 2 and 3, and lanes 5 and 6, 7). Third, when samples were analysed by gel filtration under reducing conditions, most of the protein was still eluted in the void volume. Fourth, mass spectrometric analysis of proteolytic digests of dimeric MBP-ZP-N-6his did not reveal additional peaks compared to monomeric protein, whose spectra were consistent with native, intramolecular disulfides (ZP3 Cys 1 (aa 46)-Cys 4 (aa 139) and Cys 2 (aa 78)-Cys 3 (aa 98)) (Fig. 2C, D) [3,6-10].

### Structural analysis of recombinant ZP-N

**Figure 3:**
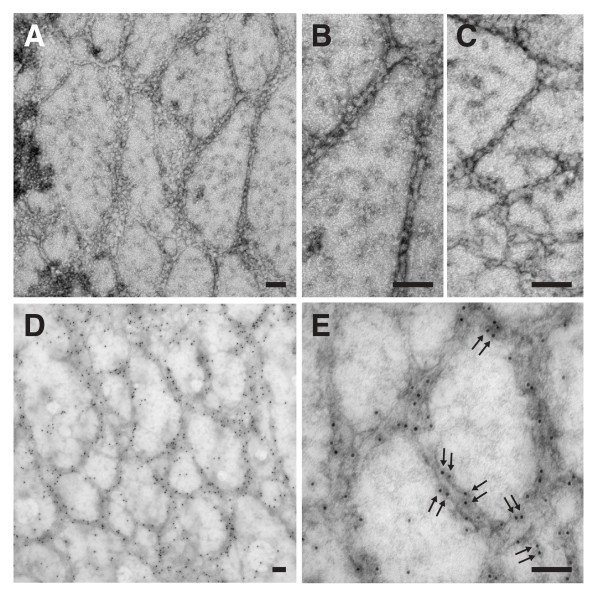
**MBP-ZP-N-6his assembles into filaments**. **(A-C) **Electron micrographs showing overview (A) and details (B, C) of negatively stained samples. **(D, E) **Immunogold localisation using monoclonal anti-MBP. Arrows mark closely spaced pairs of beads. Bars represent 0.1 μm.

Electron microscopy (EM) of negatively stained MBP-ZP-N-6his revealed that the protein assembles into long filaments (Fig. 3A) whose features are reminiscent of the helical structure described for full-length ZP domain proteins (Fig. 3B, C) [2,3]. Moreover, a pattern was observed in immunolocalisation studies which suggests that dimeric MBP-ZP-N-6his is present as repeating units within filaments (Fig. 3D, E).

## Discussion

Our results indicate that *E. coli*-expressed MBP-ZP-N-6his is correctly folded and, because MBP is monomeric and does not influence the multimerisation state of passenger proteins [21-23], that the fusion protein assembles into filaments through its ZP-N sequence. The solubility of purified MBP-ZP-N-6his filaments can be explained by the well documented solubilisation properties of MBP [27,28]. Furthermore, the periodicity observed by both SDS-PAGE (Fig. 2B) and EM (Fig. 3E) suggests that multimerisation of MBP-ZP-N-6his involves formation of non-covalently linked homodimers. Consistent with these conclusions, a large portion of ZP-C sequence is apparently missing from polymeric Tamm-Horsfall protein due to proteolytic processing between conserved Cys 6 and 7 of the ZP domain [29]. Moreover, homodimerisation of full-length ZP domain proteins, including mammalian ZP3, has been described [3,9,30-33].

By demonstrating that ZP-N is a conserved, autonomously folding unit that is biologically active, we suggest that this sequence should be considered a domain on its own and that the current definition of ZP domain should be revised. PLAC1-like proteins are able to polymerise and this explains why the majority of ZP domain mutations causing disease in humans, such as those in α-tectorin and Tamm-Horsfall protein, are clustered within the first half of the domain [3,34-36]. The importance of ZP-N is also underscored by the observation that ZP domain protein endoglin contains a canonical ZP-N sequence whereas only 2 Cys are conserved within its ZP-C subdomain ([37-40]; accession number AAT84715), and that some fish ZP1 protein isoforms completely lack ZP-C ([41]; Table 1). The availability of a recombinant ZP-N construct able to assemble into filaments that can be easily purified will be instrumental in understanding the effects of these mutations at the molecular level. Our results also raise important questions about the structure of ZP domain filaments and the function of ZP-C. Because the latter is only found as part of a complete ZP domain and can adopt different disulfide connectivities [3,6-9], it may play a crucial role in regulating the specificity of ZP-N to determine whether or not a given ZP domain protein can homo- or heteropolymerise. Indeed, presence of ZP-C, as well as of hydrophobic patches that regulate polymerisation of ZP domain proteins [4], within full-length ZP3 could explain why – unlike its ZP-N fragment – this is apparently not able to assemble into filaments in the absence of a type II ZP domain counterpart [9,42,43]. Alternatively, it is possible that full-length ZP3 and ZP2 are in principle also able to homopolymerise, but the resulting filaments are not stable unless they interact with each other [10].

## Conclusion

Recent studies led to the hypothesis that the ZP domain, a module responsible for the polymerisation of a large number of extracellular proteins, consists of two subdomains. In this work, we identified protein sequences sharing homology exclusively with the N-terminal half of the ZP domain (ZP-N), but did not find sequences containing only its C-terminal half (ZP-C). We then showed that a recombinant protein corresponding to the ZP-N region of mammalian sperm receptor ZP3 is able to fold independently from its ZP-C counterpart, and that it assembles into filaments which appear to consist of dimeric subunits. Our results argue that ZP-N should be considered a domain of its own, suggest a function for proteins containing only ZP-N, are consistent with the higher structural conservation of the N-terminal part of the ZP domain, and provide an explanation for the clustering of mutations within ZP-N. Finally, we propose that ZP-C might function by regulating ZP-N-mediated polymerisation of proteins containing a full ZP domain.

## Methods

### Sequence analysis

Calibrated profile HMMs for ZP-N and ZP-C were generated with HMMER 2.3.2 [44], using sequence databases derived from the Pfam [45] ZP domain protein family (PF00100) alignment. Sequences that were not complete within the amino acid range of interest were removed prior to HMM building. In the case of ZP-N, sequences that did not contain all conserved Cys 1–4 were also excluded, whereas conservation of Cys 5–8 was not explicitly imposed for inclusion of the more divergent ZP-C sequences. Profile HMMs were used to scan Ensembl [46] genome databases and the NCBI Entrez non-redundant protein database (~3800000 total sequences), and matching sequences were automatically extracted and submitted to BLAST [47], CD-SEARCH [48] and SMART [49]. Entries that were either partial (based on the alignment and annotation of matching BLAST sequences) or contained a complete ZP domain (as indicated by CD-SEARCH and/or SMART, as well as by their presence within both ZP-N and ZP-C matches) were filtered out, and remaining entries (~800 sequences) were individually analysed. Final acceptance criteria were high significance and completeness of the matches, as indicated by HMM E-values < 0.1 and extent of the alignment to HMM profiles (together with presence of conserved Cys 1–4 (ZP-N) or Cys 5–8 (ZP-C)), respectively. In addition, since both proteins with a complete ZP domain and PLAC1-like proteins are secreted, matches were accepted only if they also included a putative signal peptide (as predicted by SignalP [50] and EMBOSS SigCleave [51,52]) which did not overlap with ZP domain sequence (as identified by CD-SEARCH and/or SMART). This analysis yielded 8 unique sequences containing only ZP-N, and no sequences containing only ZP-C (Table 1). An additional mouse sequence with E-value = 1.2 (protein LOC225923; accession number NP_001028455.1) was added to the ZP-N protein list on the basis of its significant similarity to proteins Oosp1 and LOC219990. BLAST and BLAT [53] searches of the mouse genome indicated that the genes encoding proteins Oosp1 and LOC225923, as well as the gene for a third protein (LOC225922; accession number NP_001032723.1) homologous to human LOC219990, are closely located on chromosome 19. The same cluster was independently identified in a recent study, in which LOC225922 and LOC225923 were renamed Oosp2 and Oosp3, respectively [19].

### DNA constructs

A PCR fragment encoding aa 42–143 of mouse ZP3 protein was cloned between the *Eco*R1 and *Xho*1 sites of vector pMBP4c, a derivative of plasmid pMBPL-/gp21(338–425) [54] that expresses a C-terminally histidine-tagged modified version of MBP under the control of T7 promoter/*lac *operator. A second vector, pLJDIS1, was generated from plasmids pBADΔSSdsbC [24] and pFÅ5 [25] to allow co-expression of a version of disulfide isomerase lacking a signal sequence (ΔSSdsbC) and a glutaredoxin-like thioredoxin variant with higher redox potential (TrxA(G33P, P34Y)), under the control of the arabinose promoter. All constructs were verified by DNA sequencing.

### Protein expression and purification

For over-expression of MBP-ZP-N-6his, pMBP4c-mZP3(42–143) and pLJDIS1 were co-transformed into *E. coli *Origami B (DE3) (Novagen), carrying *trxB *and *gor *mutations. Although the *trxB gor *background was crucial to get partially soluble MBP-ZP-N-6his (the protein was completely insoluble in BL21 (DE3)), no significant improvement in solubility was observed upon co-expression of ΔSSdsbC or TrxA(G33P, P34Y). Nevertheless, we decided to still co-express both proteins, because they could be qualitatively important, as they were shown to significantly increase the activity of recombinant disulfide-rich proteins expressed in the cytoplasm of *E. coli trxB gor *strains [24]. Transformed cells were grown at 37°C in M9 medium containing 0.4% glucose, 15 μg/ml kanamycin, 12.5 μg/ml tetracyclin, 25 μg/ml chloramphenicol and 100 μg/ml carbenicillin. After reaching an optical density (OD_595 nm_) of 0.5, they were shifted to 24°C for 30 min and pre-induced with 0.2% arabinose. 1 hr 30 min later, cells were induced with 0.1 mM isopropyl-β-D-thiogalactopyranoside and grown for an additional 25 hr at 24°C (final OD_595 nm_~0.75). Bacteria were harvested by centrifugation and lysed with CelLytic B (Sigma). Soluble MBP-ZP-N-6his was purified by affinity chromatography, using Ni^2+^-charged HiTrap Chelating HP (Amersham Biosciences) and amylose resin (New England Biolabs) columns, followed by step-gradient ion exchange chromatography, using a Mono Q column (Amersham Biosciences). After dialysis against buffer F (10 mM Na-HEPES pH 8.0, 100 mM NaCl, 1 mM maltose, 1 mM NaN_3_), the purified protein was concentrated to 16 mg/ml.

### Western blotting

Immunoblot experiments were carried out by using BSA-free Penta•His monoclonal primary antibody (1:1000; QIAGEN) and goat anti-mouse horseradish peroxidase (HRP)-conjugated IgG (1:3000; ICN/Cappel), according to the manufacturers protocol. Chemiluminescent detection reactions were performed with Western Lightning Chemiluminescence Reagent *Plus *(Perkin Elmer).

### Mass spectrometry

After SDS-PAGE under non-reducing conditions (with ~20 μg MBP-ZP-N-6his/lane), gel spots were excised and alkylated with 30 mM iodoacetamide in 100 mM Tris-HCl pH 6.8 for 30 min at room temperature. The liquid was removed and samples were prepared for digestion by washing twice with 100 ml 50 mM Tris-HCl pH 6.8/30% acetonitrile (ACN) for 20 min with shaking, then with 100% ACN for 1–2 min. After removing the washes, gel pieces were dried for 30 min in a Speed-Vac concentrator. Individual gel pieces were digested by adding 80 μg modified trypsin or chymotrypsin (sequencing grade, Roche Molecular Biochemicals) in 13–15 ml 25 mM Tris-HCl pH 6.8 and leaving overnight at room temperature. Peptides were extracted with 2 × 50 ml 50% ACN/2% trifluoroacetic acid (TFA) and the combined extracts were divided in half, then dried. One half of the digest was dissolved in matrix-assisted laser desorption/ionisation time-of-flight mass spectrometry (MALDI-TOF-MS) matrix for immediate mass spectrometric analysis, and the other half was reduced by adding 20 mM dithiothreitol (DTT) in 100 mM Tris-HCl pH 8.5. After 30 min at 50°C, the reduced digest was cooled to room temperature and desalted with a C18 ZipTip (Millipore), using 50% ACN to elute the peptides. The eluate was dried and dissolved in MALDI-TOF-MS matrix for analysis. Matrix solution was prepared by making a 10 mg/ml solution of 4-hydroxy-α-cyanocinnamic acid in 50% ACN/0.1% TFA. The dried digest was dissolved in 3 ml matrix solution and 0.7 ml was spotted onto the sample plate. If the sample was not previously desalted, the dried spot was washed twice with water. MALDI mass spectrometric analysis was performed on the digest using a Voyager DE-Pro mass spectrometer (Applied Biosystems) in the linear mode. Spectra were analysed both manually and with MS-Screener [55] and MS-Compare (LJ, unpublished). Since all samples were alkylated prior to digestion, unmodified free Cys-containing peptides identified under non-reducing conditions (Fig. 2C) resulted from laser-induced breakage of disulfides. Furthermore, it appeared that essentially all Cys residues of purified MBP-ZP-N-6his were involved in disulfides. Unlike the case of the Cys 2-Cys 3 disulfide bridge (Fig. 2C), a peak corresponding to a linkage between peptides containing Cys 1 and Cys 4 could not be identified under non-reducing conditions; however, existence of the latter bridge could be clearly inferred by appearance (or marked increase in the intensity) of peaks corresponding to peptides containing unmodified free Cys 1 and Cys 4 upon reduction of the sample (compare Fig. 2C and 2D). This was further supported by a corresponding increase in the intensity of a peak corresponding to the C-terminal tag, which closely follows Cys 4 in the sequence of MBP-ZP-N-6his (Fig. 2C, D). MALDI-TOF-MS analyses of chymotrypsin-digested monomeric protein as well as trypsin-digested dimeric MBP-ZP-N-6his were also consistent with intramolecular 1–4, 2–3 disulfides.

### Size-exclusion chromatography

Gel filtration experiments were performed on both FPLC and HPLC systems, using a HiPrep 16/60 Sephacryl S-300 HR column (~300 kDa M_r _cut-off; Amersham Biosciences) and a Bio-Sil SEC-250-5 column (~300 kDa M_r _cut-off; Bio-Rad), respectively. Running solutions were buffer F (non-reducing conditions) or buffer F + 10 mM DTT (reducing conditions). Additional runs were performed by pre-incubating purified MBP-ZP-N-6his with 10 mM EDTA pH 8.0 for 1 hr at 4°C, before analysis using 10 mM Na-HEPES pH 8.0, 1 mM EDTA as running buffer.

### Electron microscopy

For morphological observation, material was negatively stained by applying a drop of solution (final concentration 1 mg/ml) directly onto a 300-mesh formvar-carbon coated nickel grid (Electron Microscopy Sciences), which was allowed to remain for approximately 30 seconds, after which excess solution was removed. A drop of 1% aqueous uranyl acetate was then added onto the grid and allowed to remain for an additional 30 seconds, after which excess solution was removed and the grids allowed to dry. For immunogold localisation, equal volumes of protein (1 mg/ml) and anti-MBP monoclonal primary antibody (1:300; New England Biolabs) diluted in Tris-buffered saline-Tween-20 solution (TBS-T) were allowed to incubate for two hours at room temperature. Goat anti-mouse H&L(Fab2') 10 nm gold-conjugated secondary antibody (1:30/TBS-T, EMS) was added directly to the solution and allowed to incubate for two hours at room temperature. A 300-mesh formvar-carbon coated nickel grid was then immersed and allowed to remain for approximately 30 seconds, after which it was removed and excess solution was removed. Negative contrast staining followed the above-described method. Material was imaged on a Jeol 1200EX electron microscope equipped with an Advanced Imaging Technologies digital camera. Images were imported into Photoshop CS2 (Adobe Systems Inc.) where they were sized and optimised for contrast and brightness.

## Abbreviations

6his: 6-histidine tag

aa: amino acid(s)

ACN: acetonitrile

DTT: dithiothreitol

EDTA: ethylenedinitrilotetraacetic acid

EM: electron microscopy

FPLC: fast protein liquid chromatography

HMM: hidden Markov model

HPLC: high performance liquid chromatography

IMAC: immobilised metal ion affinity chromatography

*m/z*: mass/charge ratio

M_r_: molecular weight

MALDI-TOF-MS: matrix-assisted laser desorption/ionisation time-of-flight mass spectrometry

MBP: maltose-binding protein

OD: optical density

SDS-PAGE: sodium dodecyl sulfate-polyacrylamide gel electrophoresis

TBS-T: Tris-buffered saline-Tween-20 solution

TFA: trifluoroacetic acid

ZP: zona pellucida

## Authors' contributions

LJ conceived the study, generated the ZP-N expression construct, purified the recombinant protein, analysed it by SDS-PAGE and size exclusion FPLC, and took part in the interpretation of mass spectrometry data. WGJ carried out the electron microscopy studies. ESL performed the size exclusion HPLC experiments. PMW participated in experimental design and data analysis. The paper was written by LJ and PMW, and has been read and approved by all the authors.
